# Non-communicable diseases and maternal health: a scoping review

**DOI:** 10.1186/s12884-022-05047-6

**Published:** 2022-10-22

**Authors:** Tabassum Firoz, Beth Pineles, Nishika Navrange, Alyssa Grimshaw, Olufemi Oladapo, Doris Chou

**Affiliations:** 1 Yale New Haven Health, Bridgeport Hospital, Bridgeport, CT USA; 2 Department of Obstetrics, Gynecology and Reproductive Sciences, McGovern Medical School, The University of Texas Health Science Center at Houston (UTHealth), Houston, TX USA; 3 New York University, New York, NY USA; 4 Harvey Cushing/John Hay Whitney Medical Library, Yale University, New Haven, CT USA; 5 World Health Organization, Geneva, Switzerland

**Keywords:** Non-communicable diseases, Maternal health, Pregnancy, Childbirth

## Abstract

**Background:**

Non-communicable diseases [NCDs] are leading causes of ill health among women of reproductive age and an increasingly important cause of maternal morbidity and mortality worldwide. Reliable data on NCDs is necessary for accurate measurement and response. However, inconsistent definitions of NCDs make reliable data collection challenging. We aimed to map the current global literature to understand how NCDs are defined, operationalized and discussed during pregnancy, childbirth and the postnatal period.

**Methods:**

For this scoping review, we conducted a comprehensive global literature search for NCDs and maternal health covering the years 2000 to 2020 in eleven electronic databases, five regional WHO databases and an exhaustive grey literature search without language restrictions. We used a charting approach to synthesize and interpret the data.

**Results:**

Only seven of the 172 included sources defined NCDs. NCDs are often defined as chronic but with varying temporality. There is a broad spectrum of conditions that is included under NCDs including pregnancy-specific conditions and infectious diseases. The most commonly included conditions are hypertension, diabetes, epilepsy, asthma, mental health conditions and malignancy. Most publications are from academic institutions in high-income countries [HICs] and focus on the pre-conception period and pregnancy. Publications from HICs discuss NCDs in the context of pre-conception care, medications, contraception, health disparities and quality of care. In contrast, publications focused on low- and middle-income countries discuss NCDs in the context of NCD prevention. They take a life cycle approach and advocate for integration of NCD and maternal health services.

**Conclusion:**

Standardising the definition and improving the articulation of care for NCDs in the maternal health setting would help to improve data collection and facilitate monitoring. It would inform the development of improved care for NCDs at the intersection with maternal health as well as through a woman's life course. Such an approach could lead to significant policy and programmatic changes with the potential corresponding impact on resource allocation.

**Supplementary Information:**

The online version contains supplementary material available at 10.1186/s12884-022-05047-6.

## Background

Globally, non-communicable diseases (NCDs) are the leading cause of death and disability in women, including in women of reproductive age [1]. The Sustainable Development agenda includes specific targets on both maternal health and NCDs. Sustainable Development Goal (SDG) Target 3.1 calls for a reduction of the global maternal mortality ratio to 70 deaths per 100,000 livebirths; and SDG 3.4 calls to reduce by one-third premature mortality from NCDs [2]. Given the growing prevalence of NCDs worldwide, the Global Strategy for Women’s, Children’s and Adolescents’ Health [2016–2030], a roadmap on ending all preventable deaths in women, integrated NCDs into the Sexual Reproductive Maternal Neonatal Child Adolescent Health (SRMNCAH) response and included a target addressing NCDs as an essential component of the *Survive* pillar [3].

Evidence of the burden of non-communicable diseases in maternal health can be found in the 2014 systematic analysis of the global causes of maternal deaths [4]. Maternal deaths are divided into direct (obstetric-related) and indirect causes by the International Classification of Diseases (ICD) [5]. Deaths due to NCDs are considered amongst the fraction of “indirect” maternal deaths. The 2014 analysis found that indirect causes contributed to the same proportion [27%] of maternal deaths as hemorrhage and a higher proportion than hypertensive disorders, which account for the majority of obstetric-related deaths [4]. Yet the approach to NCDs in pregnancy is not well articulated in the maternal health literature. The SRMNCAH continuum of care, especially pregnancy, offers critical entry points for women who may not otherwise access healthcare services. Pregnancy and the postnatal period provide crucial opportunities to integrate NCD services which would allow for early prevention, identification and management. Integrated care of NCDs in pregnancy also has the potential to improve fetal/newborn outcomes and positively impact the overall health and wellbeing for the pregnant woman, her family, and community.

In order to develop interventions that can improve the quality of care and reach of service delivery, reliable data on NCDs in pregnancy is critical. However, there is questionable reliability of the data, especially from low resource settings. Data is limited and there are large variations in the reported prevalence of NCDs in pregnancy. Prevalence data is often generated from hospital-based studies and in low resource settings, where women often cannot reach health facilities, and where health facility birth rates can be low, hospital studies are likely to underestimate the burden of disease. Although the measurement of NCDs in pregnancy is beyond the scope of this paper, inaccurate estimates of the burden of NCDs in pregnancy may result in inefficient research, prioritization and focus in this area of intersection.

A key challenge is that the data are subject to inconsistent definitions of NCDs as a standardized definition is lacking. A wide spectrum of conditions such as medical conditions, malignancies and mental health conditions with varying temporality from acute to chronic have been included under the umbrella of NCDs. It is necessary to arrive at a common understanding of NCDs for accurate and routine measurement of NCDs in order to inform policy decisions, resource allocation and ultimately to launch an appropriate programmatic response to reduce maternal morbidity and mortality.

As a first step towards this process, we undertook a scoping review to map the current literature to understand how NCDs are defined, operationalized and discussed specifically during pregnancy, childbirth and the postnatal period. We also sought to describe the disease conditions that are included within the umbrella of NCDs. Finally, we aimed to identify partners, programs and organizations working in the area of NCDs and maternal health.

## Methods

The scoping review was conducted using the framework outlined by the Joanna Briggs Institute (https://jbi.global/scoping-review-network/resources#) and reported using the Preferred Reporting Items for Systematic Reviews and Meta-Analyses (PRISMA) Extension for Scoping Reviews statement (Table S1) [6, 7]. The protocol was registered in Open Science Framework (https://archive.org/details/osf-registrations-trvzf-v1).

A literature search was conducted in African Index Medicus, Africa Wide Information, CINAHL, CKNI, Cochrane Library, Ovid Embase, Google Scholar, IMEMR [Index Medicus for Eastern Mediterriean Region], IMSEAR [Index Medicus for the South-East Asia Region], LILACS [Latin America and the Caribbean Literature on Health Sciences], Ovid Medline, PubMed, PsycInfo, Scopus, Web of Science Core Collection, and WPRIM [Western Pacific Region Index Medicus] to identify papers between 2000–2020 using a combination of controlled vocabulary and keywords for “non-communicable diseases” and “maternal health”. The year 2000 was chosen as the start date as the Millennium Development Goals (MDG) were introduced that year and specifically included a target on maternal mortality, drawing global attention to maternal health [8]. Databases were last searched on November 12, 2020. The search strategies for all databases are outlined in Table S2. A grey literature search was conducted in Google and websites of relevant organizations working in the area of NCDs and maternal health. We hand searched references of included systematic reviews. There were no language restrictions used in this study. As this was a global search, relevant data from all countries were included.

Studies were included if they focused on the population of interest which included reproductive age, pregnant or postpartum women and discussed specific disease conditions within the context of maternal health care provision. Studies of reproductive age women were included if they had implications for pregnancy care. We included studies that provided original data [e.g. observational studies, randomized controlled trials] as well as narrative reviews, commentaries, white papers, policy documents, professional society position statements, blog posts and information from websites of relevant organizations.

We excluded studies that simply provided enumeration of NCDs, focused on placental, fetal or neonatal outcomes, or on long-term consequences of NCDs and those that only reported on adverse pregnancy outcomes in women with NCDs. Studies were also excluded if they were protocols of studies without results.

Citations from all databases were imported in an Endnote × 9 library (Clarivate Analytics, Philadelphia, PA). After removing duplicates using the Yale Reference Deduplicator, the remaining set of articles was imported into Covidence (https://www.covidence.org), a screening and data extraction tool. Two authors independently screened the titles [AG and TF] and abstracts to determine which studies would undergo full-text review [BP, DC, TF and NN]. The full text of the resulting papers was then reviewed for inclusion by two authors independently [BP, DC, TF and NN]. In both screenings, discrepancies were resolved by discussion between BP, DC, and TF. Data extraction was conducted using a standardized data abstraction sheet which was pilot tested by TF and BP and revised to ensure consistency in data abstraction. We extracted data on study type, country of publication, publishing institution(s), target population, definition of NCDs, disease conditions and main findings of the source. Countries were classified using the World Bank Country and Lending Groups (https://datahelpdesk.worldbank.org/knowledgebase/articles/906519-world-bank-country-and-lending-groups). We classified lower middle and low income economies as “low resource settings”. Data was extracted by at least one of the study members [BP, NN and TF] and reviewed by a second member [DC] also in Covidence.

We aimed to synthesize a thematic framework of maternal health care provisions for NCDs and used a charting approach to synthesize and interpret the data. This technique involved the creation of a ‘data charting form’ using the database program Excel where information about the studies and outcomes was recorded and organized according to key issues and themes relevant to NCDs and maternal health.

## Results

**Fig. 1:**
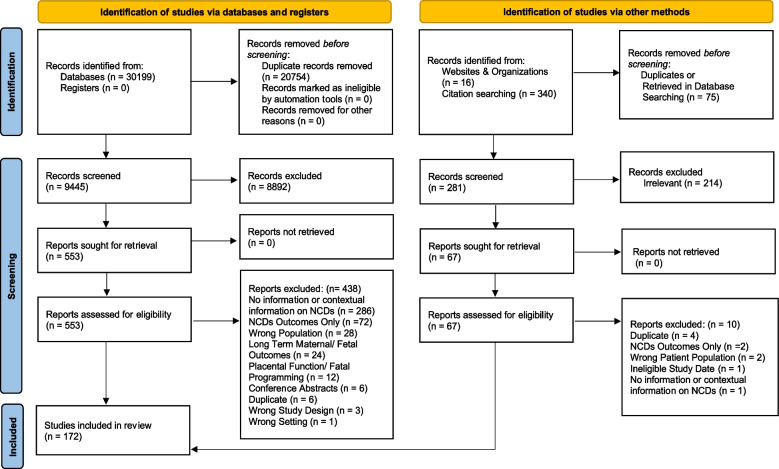
PRISMA. Adapted from: Page MJ, McKenzie JE, Bossuyt PM, Boutron I, Hoffmann TC, Mulrow CD, et al. The PRISMA 2020 statement: an updated guideline for reporting systematic reviews. BMJ 2021;372: n71. doi: 10.1136/bmj. n71. For more information, visit: http://www.prisma-statement.org/

A total of 30,199 papers were identified in the databases. After deduplication, 9,445 articles underwent initial screening by title and abstract, 553 had a full text evaluation, and a total of 115 peer-reviewed papers met all of the criteria for inclusion in this review (Fig. 1).

**Fig. 2:**
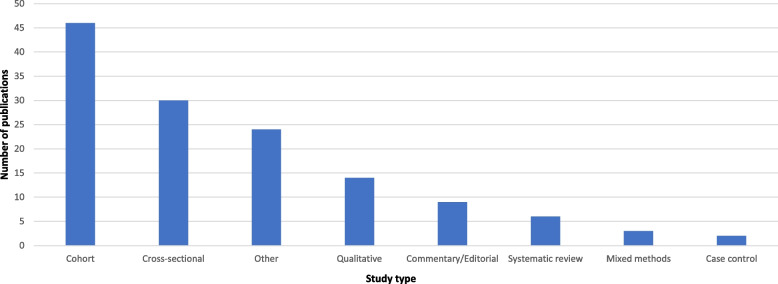
Publications by study type

An additional 41 peer-reviewed papers were identified through citation chasing and one peer-reviewer paper was identified during the gray literature search. Fifteen online sources were included from the grey literature search, 14 of which were blog posts and one was an online newspaper article. There were a total of 172 included records [9–180]. Almost half (*n* = 76) of the peer-reviewed publications were cohort and cross-sectional studies. We identified six systematic reviews,^1^ 14 qualitative studies, and 23 narrative reviews. The remaining 38 peer reviewed publications were mixed methods studies, descriptive studies, population census, secondary reports of maternal death reviews, editorials/commentaries, professional society position statements, workshop or working group reports. Figure 2 shows the publications by study type.

### Regional distribution

**Fig. 3:**
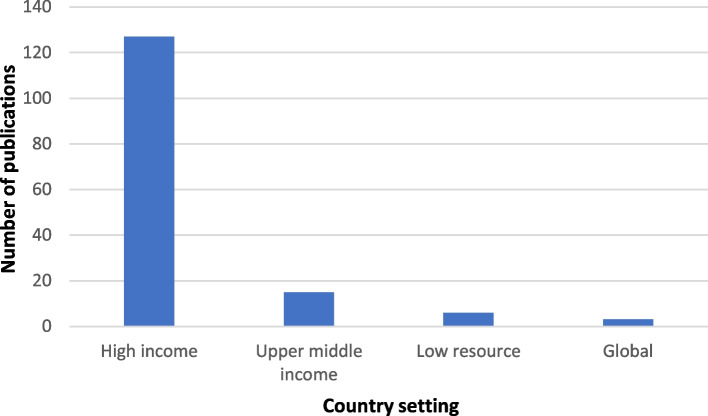
Publications by country income level

Three of the peer reviewed publications were global in nature. Of the remaining 148 peer reviewed publications, 85.8% [*n* = 127] were from high-income countries [HICs], 10.1% [*n* = 15] from upper middle-income countries and only 4.1% [*n* = 6] from low resource settings (Fig. 3). 71.6% [*n* = 106] of the peer-reviewed publications was from Europe and Northern America. 13 publications were from Oceania and 10 were from Latin America and the Caribbean. There were only 16 publications in total from the remaining SDG regions [Sub Saharan Africa, Central and Southern Asia and Eastern and South-Eastern Asia, Eastern and South-Eastern Asia and Northern Africa and Western Asia].

Many papers had authors from one region and concentrated on a population in a different region. About 15% of the peer-reviewed papers and grey literature sources concentrated specifically on populations in low resource settings. Two thirds of the peer reviewed papers focusing on low resource settings were from Europe and Northern America. Only eight of the peer reviewed publications focusing on populations in low resource settings were based on primary data [53, 88, 102, 116, 118, 131, 147, 156], with half published by HIC authors and the other half published by authors from low resource settings. Of the 15 peer reviewed publications that had a global focus, 80% were from Europe and Northern America and mainly discussed health care policy, particularly NCD prevention and service integration.

### Institutions and organizations

84.1% [*n* = 127] of peer-reviewed papers were published by academic institutions. 71.7% [*n* = 91] of these publications were from Europe and Northern America. Publications from civil societies, governmental organizations, inter-governmental organizations, and private institutions accounted for 9.9% [*n* = 15] of the publications. 6.6% [*n* = 10] were joint publications by more than one type of institution or organization. 43% of the blog posts were published by the Maternal Health Task Force, which is an US-based organization. Other organizations publishing blog posts on NCDs in maternal health include the NCD Alliance, Women Deliver, the Wilson Center and Ending Eclampsia.

### Target population^2^

**Fig. 4:**
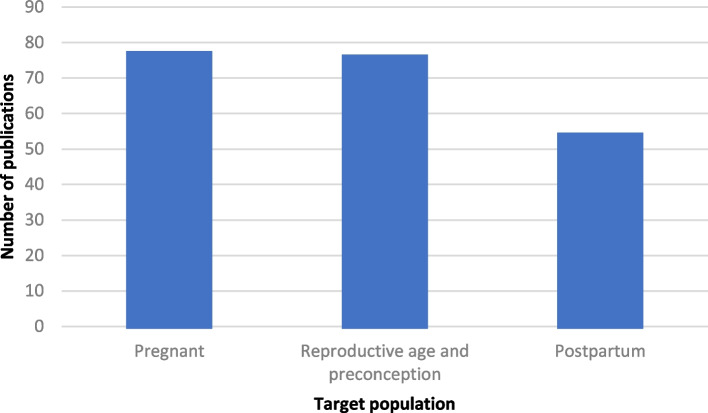
Publications by target population

About half [51%] of the peer-reviewed publications focused on pregnant women. A similar proportion of papers [50.3%] focused on pre-conception care and reproductive age women while only about a third of papers [35.8%] focused on postpartum women (Fig. 4). Grey literature sources often had more than one population of focus. All the grey literature sources focused on pregnant women with 43% also focusing on reproductive age women and only 25% focusing on postpartum women.

### Definitions

Only three of the peer-reviewed papers [25, 63, 161] and four of the blog posts [168, 169, 177, 180] contained a definition of NCDs. Often, NCDs were mostly discussed as “chronic illness”, “chronic disease” or “chronic medical problems”. One paper described NCDs as “chronic manageable” versus “long-term life-threatening conditions” Table 1 outlines the definitions of NCDs in the available maternal health literature.

**Table 1:** Varying definitions of NCDs

• Conditions were defined as *chronic* according to whether they lasted 12 months or longer and (1) limited independent living, social interactions, or self-care or (2) required *ongoing* intervention with medical services, products or equipment
• *Chronic medical condition* was defined as being recognized and treated for 3 months or more prior to pregnancy, requiring medical attention or medication, and known to be *persistent*, *incurable, and may worsen over time*
• Chronic conditions that are *treatable but seldom curable*
• Chronic conditions, each associated with *obstetric morbidity and mortality*
• NCDs, often referred to as *chronic illnesses*, are *non-transmissible* diseases that may be caused by genetic or behavioral factors, and generally have *a slow progression and long duration*
• “Silent *diseases”*; (conditions which) “cause a *gradual deterioration in health*”

### Spectrum of NCDs

A wide spectrum of conditions was included under the umbrella of NCDs. The most common conditions were hypertension [46.3%, *n* = 77 sources^3^], diabetes [74.1%, *n* = 123 sources], obesity [15%, *n* = 25 sources], mental health conditions [39.8%, *n* = 66 sources], malignancy [22.9%, *n* = 38 sources], epilepsy [21.1%, *n* = 35 sources] and asthma [23.5%, *n* = 39 sources]. Table S3 outlines conditions by organ system. The commonly included malignancies were breast and cervical cancer. The most common mental health conditions were depression and anxiety. There was a broad spectrum of mental health conditions in the literature but at the same time, several papers did not define mental health conditions. Although by definition, the following conditions are infectious diseases; they were considered in papers due to their chronic nature and other non-infectious sequelae: human immunodeficiency virus (HIV)human papilloma virus (HPV), herpes simplex virus, tuberculosis, schistosomiasis and malaria.

Papers also included pregnancy-specific conditions such as pre-eclampsia, gestational diabetes, peripartum cardiomyopathy, obstetric fistula and postpartum depression as NCDs. The most commonly mentioned risk factors for NCDs included smoking, alcohol consumption, and diet/nutrition.

### Thematic areas of NCDs

Table 2 describes the main thematic areas.

**Table 2:** Thematic areas

Thematic areas with sub-themes
Disparities
Healthcare acces and utilization
Insurance
Race/ethnicity
Socio-economic status
Health systems and service delivery
Healthcare spending
Integration of services
Programs and initiatives
Maternal mortality
Medications
Contraception
Medications usage during pregnancy and lactation
Women’s attitudes and knowledge
Mental Health
Link to chronic disease
Risk factors
Policy
Global agenda
Lifecourse approach
NCD prevention
NCD risk factors
Service integration
Pre-conception care and pregnancy planning
Provider attitudes, beliefs and knowledge
Women’s attitudes and perceptions
Service delivery
Special populations
Women’s experiences
Pregnancy and postpartum experience
Quality of life
Women’s knowledge of NCDs

Papers discussing NCDs in HICs predominantly focused on specific disease conditions such as hypertension, diabetes, epilepsy, malignancy, and mental health. These publications had a large focus on the pre-conception care of these conditions. These disease conditions were also commonly discussed in the context of medication and contraception usage, quality of care, women’s experiences and health care disparities. There were three papers from HICs focused on the pre-conception care of women over 40 [43], post-menopausal women [14] and those undergoing in vitro fertilization [146].

Publications that discussed NCDs in the context of low resource settings or with a global focus, were mainly driven by civil society, professional organizations and intergovernmental organizations. In contrast to papers focused on NCDs in HICs, these publications took a life course approach and often focused on prevention and screening of NCDs such as hypertension, diabetes and cervical cancer. These publications had a large focus on areas for action on health systems and policy, specifically advocating for the integration of SRMNCAH and NCD services. Some examples include using pre-natal clinics as “good entry points” for breast and cervical cancer screening, integrating cervical cancer screening into HIV clinics and targeting adolescents to deliver HPV vaccines.

In HIC settings, maternal mental health, such as antepartum and postpartum depression and anxiety, was often discussed in the context of other co-morbid conditions. Most publications looked at the association between medical conditions such as hypertension, diabetes, epilepsy and mental health conditions. Maternal mental health was also discussed in the context of substance use disorder and gender-based violence. Publications on maternal mental health with a global or LMIC focus were much more limited. These publications highlighted the impact of mental health conditions of maternal and perinatal outcomes and discussed the need for effective preventative and treatment strategies.

There were no studies with focus on low resource settings or a global focus that discussed women’s experiences of NCDs. There was only one study from Myanmar that looked at pregnant women’s knowledge of NCDs such as hypertension, diabetes, anemia, and nutritional needs during pregnancy [156]. On the other hand, there were publications with HIC focus that looked at pregnant women’s experiences of NCDs such as epilepsy, diabetes, chronic kidney disease, lupus and HIV and quality of life in pregnant women with rheumatic diseases and HIV. Two papers also examined the pregnancy experiences of women with disabilities [98, 154] Publications from HICs also looked at the knowledge and attitudes of reproductive age women with NCDs such as hypertension and diabetes towards pregnancy and pre-conception health as well as their experiences with reproductive health services.

Eighteen papers examined NCDs in the context of healthcare disparities. Fourteen of these papers were from the US [10, 11, 16, 21, 32, 40, 55, 56, 97, 105, 107, 119, 140, 150], two papers from Australia [54, 57] one paper each from Brazil [19] and India [88]. One of the papers was published by the Centers for Disease Control and Prevention (CDC) from the US in collaboration with the Ministry of Health in Brazil and Pan Americana Health Organization (PAHO) and looked at the association between factors like race, education and insurance status in Brazilian reproductive age women [119]. The rest of the US-based papers focused on a wide range of topics including disparities in insurance coverage, mental health service utilization, chronic disease risk factors, hospitalization in pregnant women with NCDs, medication discontinuation and sexual orientation and health care access. The two papers from Australia focused on Aboriginal women with one paper looking at pre-conception care and the other paper reporting on stakeholder input on health service planning for reproductive age Aboriginal women with NCD risk factors [54, 57]. The paper from Brazil, an upper middle income country, examined inequities in NCD indicators in reproductive age women who were beneficiaries of a government-based social welfare program [19]. The only paper from an LMIC setting was from India which looked at healthcare disparities in the context of out-of-pocket expenditure for the treatment of NCDs in women without insurance [88].

## Discussion

Our scoping review found that NCDs are not well defined in the maternal health literature. We found a limited number of studies and online sources that defined NCDs. While most defined NCDs as chronic conditions, there was variation in the time period associated with pregnancy. A wide range of conditions were considered to be NCDs including certain infectious diseases and pregnancy-specific conditions. Papers were predominantly from HICs and focused on pre-conception care and pregnancy with few papers focusing on postpartum care. We found that the approach to NCDs differed between HICs and low resource settings. Publications from HIC discussed NCDs in the context of medications, contraception, healthcare disparities, women’s experiences and quality of care. In contrast, publications focused on low resource settings discussed NCDs in the context of NCD prevention including in adolescents and many advocated for the integration of NCD and maternal health services.

Our findings confirm that there is no common definition or framework for NCDs in pregnancy. The wide range of conditions that are categorized as NCDs poses additional challenges to accurate measurement and impacts the quality of prevalence and incidence data. This has also been seen with mortality data as the 2014 systematic analysis of the global causes of maternal deaths described the phenomenon of misattribution and misclassification of maternal deaths, resulting in an underestimation of 20–90% of causes underlying maternal deaths across different settings [4]. While maternal mortality remains a global priority, maternal deaths have been described as the tip of the iceberg and maternal morbidity as the base [181]. The proportion of deaths due to NCDs in women aged 15–49 years has increased from 41% in 2008 to 51% in 2017 [182]. The pilot study conducted by the Maternal Morbidity Working Group found that medical problems had similar levels of prevalence as obstetric ones in pregnant women, and accounted for the majority of postpartum diagnoses, especially in Jamaica and Kenya [183]. One of the key findings of the group was that there is a lack of publications on maternal morbidity due to NCDs in the postpartum period, both in HICs and low resource settings.

A series of papers that mapped the global research agenda for maternal health found that research priorities in maternal health, especially in southern Asia and Sub Saharan Africa are not clearly aligned even with the main causes of obstetric deaths such as hemorrhage and hypertensive disorders of pregnancy [184]. Similarly, we found that despite NCDs being a major contributor of maternal death in low resource settings, few publications addressed NCDs and maternal health. Globally, pre-existing medical conditions accounted for almost 30% of maternal deaths in southern Asia and Sub Saharan Africa [5] yet we found only a handful of papers from these regions in our scoping review. In addition, many papers focused on low resource settings were written by authors based in HICs, highlighting the need to support local research infrastructure within low resource settings.

In our scoping review we found that the approach to NCD care during pregnancy differs between low resource settings and HICs. While papers with a global or low resource focus discussed NCDs in the context of health systems and service delivery and took a horizontal approach, papers with HIC focus took a vertical disease-based approach. Papers and blog posts focusing on low resource settings discussed the need for an integrated, comprehensive approach to maternal health across the life cycle. A 2017 comparative analysis of integrated care in HICs versus low resource settings found that in low resource settings, the focus has been more on developing specific clusters of services, communicable disease programs, or services for specific patient groups such as pregnant women, while integration in HICs focuses on better management of a broader group of people with multiple morbidities and/or with complex health needs with a focus on altering the wider system such as governance and financing [185].

In both settings, the epidemiology of maternal health is changing as there is a rising proportion of pregnant women with NCDs and many of these women often have multiple complex medical problems [4]. Maternal health is intimately and reciprocally linked to NCDs. Pre-existing conditions can increase both maternal morbidity and mortality while complications of pregnancy can increase the prevalence of chronic health conditions, influencing not only future pregnancies but also the long-term health of women. Although there are differing approaches to integrated care in HICs and low resource settings, a commonality is that maternal health is viewed separately from the overall general health of a woman. From a health systems perspective, a recognition of the linkages between NCDs and maternal health will facilitate integration of services. The rich body of research on integrated SRH and HIV programming over the last decade may provide insights on how to best implement such services. Furthermore, achieving seamless transitions of care between maternity services and primary care puts into action the concept of care through a woman’s life. Reframing the narrow focus of maternal health from obstetrical care moves maternal health from *surviving (pregnancy)* to *thriving* so that the health, well-being, and potential of women throughout their lives can be maximized.

Our scoping review emphasizes the need to bring a lens of equity to the intersection of NCDs and maternal health. We found a stark difference in equity research between HICs and low resource settings, with only two papers from low resource settings that looked at women’s experiences and healthcare disparities. A mapping analysis of the broader maternal health literature found that scant attention was placed on equity research in low resource settings [184]. Similar to our findings, the analysis found that publications from HICs targeted vulnerable groups such as ethnic minorities and people living in poverty, but there was a lack of such research in low resource settings. More research on equity is needed especially in low resource settings to build an evidence-based case for Universal Health Coverage (UHC), which is an essential component of social protection for women. Women with chronic conditions undoubtedly experience financial hardship due to the cost of health care. One of the papers from India included in our review found that the overall medical and non-medical expenses of non–communicable disease are much higher than those of other reproductive health related and communicable diseases and disabilities [88]. These findings underscore the importance of achieving UHC for maternity care, as detailed within EPMM [Ending Preventable Maternal Mortality] strategies to reduce maternal morbidity and mortality and as a critical component to improving the health of women with NCDs [186].

### Strengths and limitations

The main strength of our scoping review is that we conducted a comprehensive literature search of peer-reviewed papers and grey literature without language restrictions. While this is a vast subject area with sparse data, we attempted to characterize NCDs in the context of pregnancy care. We were able to synthesize a large body of work to develop a framework of how NCDs are currently defined, discussed and approached across a variety of settings. Our review highlights the limitations and gaps in the maternal heath literature which has research, programmatic, health care funding and policy implications.

## Conclusions

NCDs and their time frames are not well defined in the maternal health literature. As NCDs are a significant contributor to maternal morbidity and mortality, it is important to have a common understanding of NCDs for accurate measurement and surveillance and for quantification of health outcomes. This scoping review highlights the need for a broader approach to maternal health that addresses the changing epidemiology of maternal morbidity and mortality.

A paradigm shift is needed to view pregnancy within a continuum rather than as an isolated event in a woman’s life. There are multiple links between NCDs and the SRMNCAH continuum and there are significant opportunities to leverage solutions by breaking down disciplinary silos. By widening the scope of maternal health services to include NCDs as a part of pregnancy care, continuity in care and the transition from maternity care to primary care services after delivery is critical. This has profound implications for health systems and the provision of services will necessarily need to be contextualized by setting and resource availability. An integrated approach to NCDs in maternal health, however, could be an effective approach to inform the development of programs including priority setting, health policies and funding.

Our paper serves as the background for a program of work in the area of NCDs and maternal health. It will serve as a springboard for the development of guidelines on the management of NCDs in pregnancy and ultimately, identifying specific interventions for health systems. 

## Supplementary Information

**Additional file 1:** **Table S1.** Reporting guideline checklist. **Table S2.** Search Strategies. **Table S3.** Conditions by organ system.

## Data Availability

All data are within the manuscript and additional files however, additional data related to this project is available on Open Science Framework (https://archive.org/details/osf-registrations-trvzf-v1).

## Abbreviations

CDC: Centers for Disease Control and Prevention
EPMM: Ending Preventable Maternal Mortality
HICs: High income countries
HIV: Human Immunodeficiency Virus
HPV: Human Papilloma Virus
HSV: Herpes Simplex Virus
ICD: International Classification of Diseases
IMEMR: Index Medicus for Eastern Mediterriean Region
IMSEAR: Index Medicus for the South-East Asia Region
LILACS: Latin America and the Caribbean Literature on Health Sciences
NCDs: Non-communicable diseases
PAHO: Pan Americana Health Organization
SDG: Sustainable Development Goal
SRMNCAH: Sexual Reproductive Maternal Neonatal Child Adolescent Health
TB: Tuberculosis
UHC: Universal Health Coverage
US: United States
WPRIM: Western Pacific Region Index Medicus

## Footnotes

1 Systematic reviews were not included in the analyses below as we used the original studies identified through handsearching the references of the systematic reviews in these analyses. A total of 152 peer reviewed publications are included in the analyses.

2 Many papers had more than 1 target population. Therefore, numbers will not add up to 100%.

3 Sources = peer reviewed publications and grey literature.
