# Supplementary material for: Non-communicable diseases and maternal health: a scoping review
Source: BMC Pregnancy Childbirth. 2022 Oct 22;22:787. doi: 10.1186/s12884-022-05047-6 (PMC9587654; doi:10.1186/s12884-022-05047-6)
Supplement: Supplementary file 1 — Additional file 1: Table S1. Reporting guideline checklist. Table S2. Search Strategies. Table S3. Conditions by organ system. [file 12884_2022_5047_MOESM1_ESM.docx]

**Supplementary Tables**

# Table S1 Reporting Guideline Checklist

Preferred Reporting Items for Systematic reviews and Meta-Analyses extension for Scoping Reviews (PRISMA-ScR) Checklist

| **SECTION** | **ITEM** | **PRISMA-ScR CHECKLIST ITEM** | **REPORTED ON PAGE #** |
| --- | --- | --- | --- |
| **TITLE** | | | |
| Title | 1 | Identify the report as a scoping review. | 1 |
| **ABSTRACT** | | | |
| Structured summary | 2 | Provide a structured summary that includes (as applicable): background, objectives, eligibility criteria, sources of evidence, charting methods, results, and conclusions that relate to the review questions and objectives. | 2-3 |
| **INTRODUCTION** | | | |
| Rationale | 3 | Describe the rationale for the review in the context of what is already known. Explain why the review questions/objectives lend themselves to a scoping review approach. | 4-5 |
| Objectives | 4 | Provide an explicit statement of the questions and objectives being addressed with reference to their key elements (e.g., population or participants, concepts, and context) or other relevant key elements used to conceptualize the review questions and/or objectives. | 5 |
| **METHODS** | | | |
| Protocol and registration | 5 | Indicate whether a review protocol exists; state if and where it can be accessed (e.g., a Web address); and if available, provide registration information, including the registration number. | 5 |
| Eligibility criteria | 6 | Specify characteristics of the sources of evidence used as eligibility criteria (e.g., years considered, language, and publication status), and provide a rationale. | 6 |
| Information sources* | 7 | Describe all information sources in the search (e.g., databases with dates of coverage and contact with authors to identify additional sources), as well as the date the most recent search was executed. | 5-6 |
| Search | 8 | Present the full electronic search strategy for at least 1 database, including any limits used, such that it could be repeated. | 5-6, Table S2 |
| Selection of sources of evidence† | 9 | State the process for selecting sources of evidence (i.e., screening and eligibility) included in the scoping review. | 6-7 |
| Data charting process‡ | 10 | Describe the methods of charting data from the included sources of evidence (e.g., calibrated forms or forms that have been tested by the team before their use, and whether data charting was done independently or in duplicate) and any processes for obtaining and confirming data from investigators. | 6-7 |
| Data items | 11 | List and define all variables for which data were sought and any assumptions and simplifications made. | 7 |
| Critical appraisal of individual sources of evidence§ | 12 | If done, provide a rationale for conducting a critical appraisal of included sources of evidence; describe the methods used and how this information was used in any data synthesis (if appropriate). | N/A |
| Synthesis of results | 13 | Describe the methods of handling and summarizing the data that were charted. | 7 |
| **RESULTS** | | | |
| Selection of sources of evidence | 14 | Give numbers of sources of evidence screened, assessed for eligibility, and included in the review, with reasons for exclusions at each stage, ideally using a flow diagram. | 7 |
| Characteristics of sources of evidence | 15 | For each source of evidence, present characteristics for which data were charted and provide the citations. | 7 |
| Critical appraisal within sources of evidence | 16 | If done, present data on critical appraisal of included sources of evidence (see item 12). | N/A |
| Results of individual sources of evidence | 17 | For each included source of evidence, present the relevant data that were charted that relate to the review questions and objectives. | 7-12 |
| Synthesis of results | 18 | Summarize and/or present the charting results as they relate to the review questions and objectives. | 7-12 |
| **DISCUSSION** | | | |
| Summary of evidence | 19 | Summarize the main results (including an overview of concepts, themes, and types of evidence available), link to the review questions and objectives, and consider the relevance to key groups. | 12-15 |
| Limitations | 20 | Discuss the limitations of the scoping review process. | 15 |
| Conclusions | 21 | Provide a general interpretation of the results with respect to the review questions and objectives, as well as potential implications and/or next steps. | 16 |
| **FUNDING** | | | |
| Funding | 22 | Describe sources of funding for the included sources of evidence, as well as sources of funding for the scoping review. Describe the role of the funders of the scoping review. | 17 |

JBI = Joanna Briggs Institute; PRISMA-ScR = Preferred Reporting Items for Systematic reviews and Meta-Analyses extension for Scoping Reviews.

* Where *sources of evidence* (see second footnote) are compiled from, such as bibliographic databases, social media platforms, and Web sites.

† A more inclusive/heterogeneous term used to account for the different types of evidence or data sources (e.g., quantitative and/or qualitative research, expert opinion, and policy documents) that may be eligible in a scoping review as opposed to only studies. This is not to be confused with *information sources* (see first footnote).

‡ The frameworks by Arksey and O’Malley (6) and Levac and colleagues (7) and the JBI guidance (4, 5) refer to the process of data extraction in a scoping review as data charting*.*

§ The process of systematically examining research evidence to assess its validity, results, and relevance before using it to inform a decision. This term is used for items 12 and 19 instead of "risk of bias" (which is more applicable to systematic reviews of interventions) to include and acknowledge the various sources of evidence that may be used in a scoping review (e.g., quantitative and/or qualitative research, expert opinion, and policy document).

*From:* Tricco AC, Lillie E, Zarin W, O'Brien KK, Colquhoun H, Levac D, et al. PRISMA Extension for Scoping Reviews (PRISMAScR): Checklist and Explanation. Ann Intern Med. 2018;169:467–473. [doi: 10.7326/M18-0850](http://annals.org/aim/fullarticle/2700389/prisma-extension-scoping-reviews-prisma-scr-checklist-explanation).

Table S2 Search Strategies

The search was run by a medical librarian (AG) on November 12, 2020. A second librarian peer-reviewed the search strategies using Peer Review for Electronic Search Strategies (McGowan et. al., 2015). The Google Scholar search was performed according to Braum method were number of records in Ovid Medline influences how many results are downloaded from Google Scholar (2017).

Bramer, Wichor M., et al. "Optimal database combinations for literature searches in systematic reviews: a prospective exploratory study." Systematic reviews 6.1 (2017): 245.

McGowan, Jessie, et al. "PRESS peer review of electronic search strategies: 2015 guideline statement." Journal of clinical epidemiology 75 (2016): 40-46.

**Search for Ovid Embase**

1 exp pregnancy complication/

2 exp maternal health service/

3 exp perinatal care/

4 ((preconception or prenatal or postnatal or postpartum or maternal or perinatal or post partum or post natal or antenatal or trimester* or intrapartum) adj3 (care* or service* or health)).tw,kw.

5 (pregnan* adj1 (complication* or adverse event* or adverse effect*)).tw,kw.

6 (still birth* or stillbirth* or chorea gavidarum or pregnancy associated choreoathetos* or pregnancy induced chorea* or gestational diabetes or pregnancy induced diabetes or pregnancy induced hypertension or pregnancy transient hypertension or eclampsia* or pre-eclampsia* or HELLP Syndrome or Hemolysis Elevated Liver Enzymes Lowered Platelets Syndrome or pregnancy toxemia* or Edema Proteinuria Hypertension Gestosis or nuchal cord or fetal cord entanglement* or ectopic pregnanc* or puerperal disorder* or placenta disease*).tw,kw.

7 ((fetal or embryo or maternal) adj1 (loss or death* or mortalit*)).tw,kw.

8 (abort* or miscarr*).tw,kw.

9 or/1-8

10 exp non communicable disease/

11 exp chronic disease/

12 (non communicable disease* or non-communicable disease or noncommunicable disease or non-infectious disease* or noninfectious disease* or non infectious disease* or chronic disease* or pre-existing health condition* or pre-existing condition* or preexisting health condition* or preexisting condition* or medical disorder*).tw,kw.

13 or/10-12

14 9 and 13

15 exp animal/

16 exp animal/ and exp human/

17 15 not 16

18 14 not 17

19 limit 18 to yr="2000 -Current"

**Search for Ovid MEDLINE**

1 exp Pregnancy Complications/

2 exp Maternal Health Services/

3 exp Perinatal Care/

4 ((preconception or prenatal or postnatal or postpartum or maternal or perinatal or post partum or post natal or antenatal or trimester* or intrapartum) adj3 (care* or service* or health)).tw,kf.

5 (pregnan* adj1 (complication* or adverse event* or adverse effect*)).tw,kf.

6 (still birth* or stillbirth* or chorea gavidarum or pregnancy associated choreoathetos* or pregnancy induced chorea* or gestational diabetes or pregnancy induced diabetes or pregnancy induced hypertension or pregnancy transient hypertension or eclampsia* or pre-eclampsia* or HELLP Syndrome or Hemolysis Elevated Liver Enzymes Lowered Platelets Syndrome or pregnancy toxemia* or Edema Proteinuria Hypertension Gestosis or nuchal cord or fetal cord entanglement* or ectopic pregnanc* or puerperal disorder* or placenta disease*).tw,kf.

7 ((fetal or embryo or maternal) adj1 (loss or death* or mortalit*)).tw,kf.

8 (abort* or miscarr*).tw,kf.

9 or/1-8 590793

10 exp Noncommunicable Diseases/

11 exp Chronic Disease/

12 (non communicable disease* or non-communicable disease or noncommunicable disease or non-infectious disease* or noninfectious disease* or non infectious disease* or chronic disease* or pre-existing health condition* or pre-existing condition* or preexisting health condition* or preexisting condition* or medical disorder*).tw,kf.

13 10 or 11 or 12

14 9 and 13

15 exp animals/

16 exp animals/ and exp humans/

17 15 not 16

18 14 not 17

18 13 and 17

19 limit 18 to yr="2000 -Current"

**Search for African Index Medicus**

(tw:("non communicable disease*" or "non-communicable disease" or “noncommunicable disease” or "non-infectious disease*" or "noninfectious disease*" or "non infectious disease*" or "chronic disease*" or "pre-existing health condition*" or "pre-existing condition*" or "preexisting health condition*" or "preexisting condition*" or "medical disorder*"))) AND (tw:(Pregnan* complication* or pregnan* adverse reaction* or pregnan* adverse effect* or fetal loss or fetal embryo loss or fetal death or embryo death or maternal mortalit* or maternal death or preconception care* OR prenatal care* OR postnatal care* OR postpartum care* OR maternal care* OR perinatal care* OR "post partum" care* OR "post natal" care* OR antenatal care* OR trimester* care* OR intrapartum care* OR preconception service* OR prenatal service* OR postnatal service* OR postpartum service* OR maternal service* OR perinatal service* OR "post partum" service* OR "post natal" service* OR antenatal service* OR trimester* service* OR intrapartum service* or preconception health OR prenatal health OR postnatal health OR postpartum health OR maternal health OR perinatal health OR "post partum" OR "post natal" health OR antenatal health OR trimester* health OR intrapartum health or ("still birth*" or stillbirth* or "chorea gavidarum" or "pregnancy associated choreoathetos*" or "pregnancy induced chorea*" or "gestational diabetes" or "pregnancy induced diabetes" or "pregnancy induced hypertension" or "pregnancy transient hypertension" or eclampsia* or pre-eclampsia* or "HELLP Syndrome" or "Hemolysis Elevated Liver Enzymes Lowered Platelets Syndrome" or "pregnancy toxemia*" or "Edema Proteinuria Hypertension Gestosis" or "nuchal cord" or "fetal cord entanglement*" or "ectopic pregnanc*" or "puerperal disorder*" or "placenta disease*"))

Year Range: 2000, 2020

**Search for African-Wide Information**

TX ( ("non communicable disease*" or "non-communicable disease" or “noncommunicable disease” or "non-infectious disease*" or "noninfectious disease*" or "non infectious disease*" or "chronic disease*" or "pre-existing health condition*" or "pre-existing condition*" or "preexisting health condition*" or "preexisting condition*" or "medical disorder*" ) AND TX ( Pregnan* complication* or pregnan* adverse reaction* or pregnan* adverse effect* or fetal loss or fetal embryo loss or fetal death or embryo death or maternal mortalit* or maternal death or preconception care* OR prenatal care* OR postnatal care* OR postpartum care* OR maternal care* OR perinatal care* OR "post partum" care* OR "post natal" care* OR antenatal care* OR trimester* care* OR intrapartum care* OR preconception service* OR prenatal service* OR postnatal service* OR postpartum service* OR maternal service* OR perinatal service* OR "post partum" service* OR "post natal" service* OR antenatal service* OR trimester* service* OR intrapartum service* or preconception health OR prenatal health OR postnatal health OR postpartum health OR maternal health OR perinatal health OR "post partum" OR "post natal" health OR antenatal health OR trimester* health OR intrapartum health ) OR ( ("still birth*" or stillbirth* or "chorea gavidarum" or "pregnancy associated choreoathetos*" or "pregnancy induced chorea*" or "gestational diabetes" or "pregnancy induced diabetes" or "pregnancy induced hypertension" or "pregnancy transient hypertension" or eclampsia* or pre-eclampsia* or "HELLP Syndrome" or "Hemolysis Elevated Liver Enzymes Lowered Platelets Syndrome" or "pregnancy toxemia*" or "Edema Proteinuria Hypertension Gestosis" or "nuchal cord" or "fetal cord entanglement*" or "ectopic pregnanc*" or "puerperal disorder*" or "placenta disease*" )

Published Date: 20000101-20201120

**Search for CINAHL Complete**

TX ( ("non communicable disease*" or "non-communicable disease" or “noncommunicable disease” or "non-infectious disease*" or "noninfectious disease*" or "non infectious disease*" or "chronic disease*" or "pre-existing health condition*" or "pre-existing condition*" or "preexisting health condition*" or "preexisting condition*" or "medical disorder*" ) AND TX ( Pregnan* complication* or pregnan* adverse reaction* or pregnan* adverse effect* or fetal loss or fetal embryo loss or fetal death or embryo death or maternal mortalit* or maternal death or preconception care* OR prenatal care* OR postnatal care* OR postpartum care* OR maternal care* OR perinatal care* OR "post partum" care* OR "post natal" care* OR antenatal care* OR trimester* care* OR intrapartum care* OR preconception service* OR prenatal service* OR postnatal service* OR postpartum service* OR maternal service* OR perinatal service* OR "post partum" service* OR "post natal" service* OR antenatal service* OR trimester* service* OR intrapartum service* or preconception health OR prenatal health OR postnatal health OR postpartum health OR maternal health OR perinatal health OR "post partum" OR "post natal" health OR antenatal health OR trimester* health OR intrapartum health ) OR ( ("still birth*" or stillbirth* or "chorea gavidarum" or "pregnancy associated choreoathetos*" or "pregnancy induced chorea*" or "gestational diabetes" or "pregnancy induced diabetes" or "pregnancy induced hypertension" or "pregnancy transient hypertension" or eclampsia* or pre-eclampsia* or "HELLP Syndrome" or "Hemolysis Elevated Liver Enzymes Lowered Platelets Syndrome" or "pregnancy toxemia*" or "Edema Proteinuria Hypertension Gestosis" or "nuchal cord" or "fetal cord entanglement*" or "ectopic pregnanc*" or "puerperal disorder*" or "placenta disease*" )

Published Date: 20000101-20201120

**Search CKNI**

Title, Keyword, Abstract “pregnancy complication” Fuzzy AND Title, Keyword, Abstract “noncommunicable disease*” Fuzzy OR

Title, Keyword, Abstract “maternal heatlh” Fuzzy AND Title, Keyword, Abstract “noncommunicable disease*” Fuzzy

Used Publication Year Filter: 2000-2020

**Search for Cochrane Library**

#1 ((preconception or prenatal or postnatal or postpartum or maternal or perinatal or "post partum" or "post natal" or antenatal or trimester* or intrapartum) near/3 (care* or service* or health)):ti,ab or (pregnan* near/1 (complication* or "adverse event*" or "adverse effect*")):ti,ab or ("still birth*" or stillbirth* or "chorea gavidarum" or "pregnancy associated choreoathetos*" or "pregnancy induced chorea*" or "gestational diabetes" or "pregnancy induced diabetes" or "pregnancy induced hypertension" or "pregnancy transient hypertension" or eclampsia* or pre-eclampsia* or "HELLP Syndrome" or "Hemolysis Elevated Liver Enzymes Lowered Platelets Syndrome" or "pregnancy toxemia*" or "Edema Proteinuria Hypertension Gestosis" or "nuchal cord" or "fetal cord entanglement*" or "ectopic pregnanc*" or "puerperal disorder*" or "placenta disease*"):ti,ab or ((fetal or embryo or maternal) near/1 (loss or death* or mortalit*)):ti,ab or (abort* or miscarr*):ti,ab

#2 ("non communicable disease*" or "non-communicable disease" or “noncommunicable disease” or "non-infectious disease*" or "noninfectious disease*" or "non infectious disease*" or "chronic disease*" or "pre-existing health condition*" or "pre-existing condition*" or "preexisting health condition*" or "preexisting condition*" or "medical disorder*"):ti,ab

#3 #1 and #2

Custom Range: 01/01/2000-11/20/2020

**Search for Google Scholar**

Non communicable disease maternal health

**Search for IMEMR**

(tw:("non communicable disease*" or "non-communicable disease" or “noncommunicable disease” or "non-infectious disease*" or "noninfectious disease*" or "non infectious disease*" or "chronic disease*" or "pre-existing health condition*" or "pre-existing condition*" or "preexisting health condition*" or "preexisting condition*" or "medical disorder*"))) AND (tw:(Pregnan* complication* or pregnan* adverse reaction* or pregnan* adverse effect* or fetal loss or fetal embryo loss or fetal death or embryo death or maternal mortalit* or maternal death or preconception care* OR prenatal care* OR postnatal care* OR postpartum care* OR maternal care* OR perinatal care* OR "post partum" care* OR "post natal" care* OR antenatal care* OR trimester* care* OR intrapartum care* OR preconception service* OR prenatal service* OR postnatal service* OR postpartum service* OR maternal service* OR perinatal service* OR "post partum" service* OR "post natal" service* OR antenatal service* OR trimester* service* OR intrapartum service* or preconception health OR prenatal health OR postnatal health OR postpartum health OR maternal health OR perinatal health OR "post partum" OR "post natal" health OR antenatal health OR trimester* health OR intrapartum health or ("still birth*" or stillbirth* or "chorea gavidarum" or "pregnancy associated choreoathetos*" or "pregnancy induced chorea*" or "gestational diabetes" or "pregnancy induced diabetes" or "pregnancy induced hypertension" or "pregnancy transient hypertension" or eclampsia* or pre-eclampsia* or "HELLP Syndrome" or "Hemolysis Elevated Liver Enzymes Lowered Platelets Syndrome" or "pregnancy toxemia*" or "Edema Proteinuria Hypertension Gestosis" or "nuchal cord" or "fetal cord entanglement*" or "ectopic pregnanc*" or "puerperal disorder*" or "placenta disease*"))

Year Range: 2000, 2020

**Search for IMSEAR**

(tw:("non communicable disease*" or "non-communicable disease" or “noncommunicable disease” or "non-infectious disease*" or "noninfectious disease*" or "non infectious disease*" or "chronic disease*" or "pre-existing health condition*" or "pre-existing condition*" or "preexisting health condition*" or "preexisting condition*" or "medical disorder*"))) AND (tw:(Pregnan* complication* or pregnan* adverse reaction* or pregnan* adverse effect* or fetal loss or fetal embryo loss or fetal death or embryo death or maternal mortalit* or maternal death or preconception care* OR prenatal care* OR postnatal care* OR postpartum care* OR maternal care* OR perinatal care* OR "post partum" care* OR "post natal" care* OR antenatal care* OR trimester* care* OR intrapartum care* OR preconception service* OR prenatal service* OR postnatal service* OR postpartum service* OR maternal service* OR perinatal service* OR "post partum" service* OR "post natal" service* OR antenatal service* OR trimester* service* OR intrapartum service* or preconception health OR prenatal health OR postnatal health OR postpartum health OR maternal health OR perinatal health OR "post partum" OR "post natal" health OR antenatal health OR trimester* health OR intrapartum health or ("still birth*" or stillbirth* or "chorea gavidarum" or "pregnancy associated choreoathetos*" or "pregnancy induced chorea*" or "gestational diabetes" or "pregnancy induced diabetes" or "pregnancy induced hypertension" or "pregnancy transient hypertension" or eclampsia* or pre-eclampsia* or "HELLP Syndrome" or "Hemolysis Elevated Liver Enzymes Lowered Platelets Syndrome" or "pregnancy toxemia*" or "Edema Proteinuria Hypertension Gestosis" or "nuchal cord" or "fetal cord entanglement*" or "ectopic pregnanc*" or "puerperal disorder*" or "placenta disease*"))

Year Range: 2000, 2020

**Search for LILACS**

(tw:("non communicable disease*" or "non-communicable disease" or “noncommunicable disease” or "non-infectious disease*" or "noninfectious disease*" or "non infectious disease*" or "chronic disease*" or "pre-existing health condition*" or "pre-existing condition*" or "preexisting health condition*" or "preexisting condition*" or "medical disorder*"))) AND (tw:(Pregnan* complication* or pregnan* adverse reaction* or pregnan* adverse effect* or fetal loss or fetal embryo loss or fetal death or embryo death or maternal mortalit* or maternal death or preconception care* OR prenatal care* OR postnatal care* OR postpartum care* OR maternal care* OR perinatal care* OR "post partum" care* OR "post natal" care* OR antenatal care* OR trimester* care* OR intrapartum care* OR preconception service* OR prenatal service* OR postnatal service* OR postpartum service* OR maternal service* OR perinatal service* OR "post partum" service* OR "post natal" service* OR antenatal service* OR trimester* service* OR intrapartum service* or preconception health OR prenatal health OR postnatal health OR postpartum health OR maternal health OR perinatal health OR "post partum" OR "post natal" health OR antenatal health OR trimester* health OR intrapartum health or ("still birth*" or stillbirth* or "chorea gavidarum" or "pregnancy associated choreoathetos*" or "pregnancy induced chorea*" or "gestational diabetes" or "pregnancy induced diabetes" or "pregnancy induced hypertension" or "pregnancy transient hypertension" or eclampsia* or pre-eclampsia* or "HELLP Syndrome" or "Hemolysis Elevated Liver Enzymes Lowered Platelets Syndrome" or "pregnancy toxemia*" or "Edema Proteinuria Hypertension Gestosis" or "nuchal cord" or "fetal cord entanglement*" or "ectopic pregnanc*" or "puerperal disorder*" or "placenta disease*"))

Year Range: 2000, 2020

**Search for PubMed**

((Pregnan* complication*[Title/Abstract] OR pregnan* adverse reaction*[Title/Abstract] OR pregnan* adverse effect*[Title/Abstract] OR fetal loss[Title/Abstract] OR fetal embryo loss[Title/Abstract] OR fetal death[Title/Abstract] OR embryo death[Title/Abstract] OR maternal mortalit*[Title/Abstract] OR maternal death[Title/Abstract] OR preconception care*[Title/Abstract] OR prenatal care*[Title/Abstract] OR postnatal care*[Title/Abstract] OR postpartum care*[Title/Abstract] OR maternal care*[Title/Abstract] OR perinatal care*[Title/Abstract] OR "post partum" care*[Title/Abstract] OR "post natal" care*[Title/Abstract] OR antenatal care*[Title/Abstract] OR trimester* care*[Title/Abstract] OR intrapartum care*[Title/Abstract] OR preconception service*[Title/Abstract] OR prenatal service*[Title/Abstract] OR postnatal service*[Title/Abstract] OR postpartum service*[Title/Abstract] OR maternal service*[Title/Abstract] OR perinatal service*[Title/Abstract] OR "post partum" service*[Title/Abstract] OR "post natal" service*[Title/Abstract] OR antenatal service*[Title/Abstract] OR trimester* service*[Title/Abstract] OR intrapartum service*[Title/Abstract] OR preconception health[Title/Abstract] OR prenatal health[Title/Abstract] OR postnatal health[Title/Abstract] OR postpartum health[Title/Abstract] OR maternal health[Title/Abstract] OR perinatal health[Title/Abstract] OR "post partum"[Title/Abstract] OR "post natal"[Title/Abstract] OR "post natal" health[Title/Abstract] OR antenatal health[Title/Abstract] OR trimester* health[Title/Abstract] intrapartum health) OR ("still birth*"[Title/Abstract] OR stillbirth*[Title/Abstract] OR "chorea gavidarum"[Title/Abstract] OR "pregnancy associated choreoathetos*"[Title/Abstract] OR "pregnancy induced chorea*"[Title/Abstract] OR "gestational diabetes"[Title/Abstract] OR "pregnancy induced diabetes"[Title/Abstract] OR "pregnancy induced hypertension"[Title/Abstract] OR "pregnancy transient hypertension"[Title/Abstract] OR eclampsia*[Title/Abstract] OR pre-eclampsia*[Title/Abstract] OR "HELLP Syndrome"[Title/Abstract] OR "Hemolysis Elevated Liver Enzymes Lowered Platelets Syndrome"[Title/Abstract] OR "pregnancy toxemia*"[Title/Abstract] OR "Edema Proteinuria Hypertension Gestosis"[Title/Abstract] OR "nuchal cord"[Title/Abstract] OR "fetal cord entanglement*"[Title/Abstract] OR "ectopic pregnanc*"[Title/Abstract] OR "puerperal disorder*"[Title/Abstract] OR "placenta disease*"[Title/Abstract])) AND ("non communicable disease*"[Title/Abstract] OR "non-communicable disease"[Title/Abstract] OR "noncommunicable disease"[Title/Abstract] OR "non-infectious disease*"[Title/Abstract] OR "noninfectious disease*"[Title/Abstract] OR "non infectious disease*"[Title/Abstract] OR "chronic disease*"[Title/Abstract] OR "pre-existing health condition*"[Title/Abstract] OR "pre-existing condition*"[Title/Abstract] OR "preexisting health condition*"[Title/Abstract] OR "preexisting condition*"[Title/Abstract] OR "medical disorder*"[Title/Abstract])

Filters: from 2000 - 2022

**Search for Ovid PsycInfo**

1 exp Obstetrical Complications/

2 exp Prenatal Care/

3 exp Perinatal Period/

4 ((preconception or prenatal or postnatal or postpartum or maternal or perinatal or post partum or post natal or antenatal or trimester* or intrapartum) adj3 (care* or service* or health)).mp.

5 (pregnan* adj1 (complication* or adverse event* or adverse effect*)).mp.

6 (still birth* or stillbirth* or chorea gavidarum or pregnancy associated choreoathetos* or pregnancy induced chorea* or gestational diabetes or pregnancy induced diabetes or pregnancy induced hypertension or pregnancy transient hypertension or eclampsia* or pre-eclampsia* or HELLP Syndrome or Hemolysis Elevated Liver Enzymes Lowered Platelets Syndrome or pregnancy toxemia* or Edema Proteinuria Hypertension Gestosis or nuchal cord or fetal cord entanglement* or ectopic pregnanc* or puerperal disorder* or placenta disease*).mp.

7 ((fetal or embryo or maternal) adj1 (loss or death* or mortalit*)).mp. 1

8 (abort* or miscarr*).mp.

9 or/1-8

10 exp Chronic Illness/

11 (non communicable disease* or non-communicable disease or noncommunicable disease or non-infectious disease* or noninfectious disease* or non infectious disease* or chronic disease* or pre-existing health condition* or pre-existing condition* or preexisting health condition* or preexisting condition* or medical disorder*).mp.

12 10 or 11

13 9 and 12

14 limit 13 to yr="2000 -Current"

**Search for Scopus**

( TITLE-ABS-KEY ( ( preconception OR prenatal OR postnatal OR postpartum OR maternal OR perinatal OR "post partum" OR "post natal" OR antenatal OR trimester* OR intrapartum ) W/3 ( care* OR service* OR health ) ) OR TITLE-ABS-KEY ( pregnan* W/1 ( complication* OR "adverse event*" OR "adverse effect*" ) ) OR TITLE-ABS-KEY ( "still birth*" OR stillbirth* OR "chorea gavidarum" OR "pregnancy associated choreoathetos*" OR "pregnancy induced chorea*" OR "gestational diabetes" OR "pregnancy induced diabetes" OR "pregnancy induced hypertension" OR "pregnancy transient hypertension" OR eclampsia* OR pre-eclampsia* OR "HELLP Syndrome" OR "Hemolysis Elevated Liver Enzymes Lowered Platelets Syndrome" OR "pregnancy toxemia*" OR "Edema Proteinuria Hypertension Gestosis" OR "nuchal cord" OR "fetal cord entanglement*" OR "ectopic pregnanc*" OR "puerperal disorder*" OR "placenta disease*" ) OR TITLE-ABS-KEY ( ( fetal OR embryo OR maternal ) W/1 ( loss OR death* OR mortalit* ) ) OR TITLE-ABS-KEY ( abort* OR miscarr* ) ) AND ( TITLE-ABS-KEY ( "non communicable disease*" OR "non-communicable disease" OR “noncommunicable disease” OR "non-infectious disease*" OR "noninfectious disease*" OR "non infectious disease*" OR "chronic disease*" OR "pre-existing health condition*" OR "pre-existing condition*" OR "preexisting health condition*" OR "preexisting condition*" OR "medical disorder*" ) ) ) AND ( LIMIT-TO ( PUBYEAR , 2013 ) OR LIMIT-TO ( PUBYEAR , 2020 ) OR LIMIT-TO ( PUBYEAR , 2019 ) OR LIMIT-TO ( PUBYEAR , 2018 ) OR LIMIT-TO ( PUBYEAR , 2017 ) OR LIMIT-TO ( PUBYEAR , 2016 ) OR LIMIT-TO ( PUBYEAR , 2015 ) OR LIMIT-TO ( PUBYEAR , 2014 ) OR LIMIT-TO ( PUBYEAR , 2012 ) OR LIMIT-TO ( PUBYEAR , 2011 ) OR LIMIT-TO ( PUBYEAR , 2010 ) OR LIMIT-TO ( PUBYEAR , 2009 ) OR LIMIT-TO ( PUBYEAR , 2008 ) OR LIMIT-TO ( PUBYEAR , 2007 ) OR LIMIT-TO ( PUBYEAR , 2006 ) OR LIMIT-TO ( PUBYEAR , 2005 ) OR LIMIT-TO ( PUBYEAR , 2004 ) OR LIMIT-TO ( PUBYEAR , 2003 ) OR LIMIT-TO ( PUBYEAR , 2002 ) OR LIMIT-TO ( PUBYEAR , 2001 ) OR LIMIT-TO ( PUBYEAR , 2000 ) )

**Search for Web of Science Core Collection**

1 TS=((preconception or prenatal or postnatal or postpartum or maternal or perinatal or "post partum" or "post natal" or antenatal or trimester* or intrapartum) near/3 (care* or service* or health)) or TS=

(pregnan* near/1 (complication* or "adverse event*" or "adverse effect*")) or TS=

("still birth*" or stillbirth* or "chorea gavidarum" or "pregnancy associated choreoathetos*" or "pregnancy induced chorea*" or "gestational diabetes" or "pregnancy induced diabetes" or "pregnancy induced hypertension" or "pregnancy transient hypertension" or eclampsia* or pre-eclampsia* or "HELLP Syndrome" or "Hemolysis Elevated Liver Enzymes Lowered Platelets Syndrome" or "pregnancy toxemia*" or "Edema Proteinuria Hypertension Gestosis" or "nuchal cord" or "fetal cord entanglement*" or "ectopic pregnanc*" or "puerperal disorder*" or "placenta disease*") or TS=((fetal or embryo or maternal) near/1 (loss or death* or mortalit*)) or TS=(abort* or miscarr*)

2 TS=("non communicable disease*" or "non-communicable disease" or “noncommunicable disease” or "non-infectious disease*" or "noninfectious disease*" or "non infectious disease*" or "chronic disease*" or "pre-existing health condition*" or "pre-existing condition*" or "preexisting health condition*" or "preexisting condition*" or "medical disorder*")

3 #2 AND #1

Refined By:Publication Years: 2020 or 2018 or 2019 or 2017 or 2016 or 2015 or 2014 or 2013 or 2012 or 2011 or 2009 or 2010 or 2008 or 2007 or 2001 or 2002 or 2003 or 2004 or 2005 or 2006 or 2000

**Search for WPRIM**

(tw:("non communicable disease*" or "non-communicable disease" or “noncommunicable disease” or "non-infectious disease*" or "noninfectious disease*" or "non infectious disease*" or "chronic disease*" or "pre-existing health condition*" or "pre-existing condition*" or "preexisting health condition*" or "preexisting condition*" or "medical disorder*"))) AND (tw:(Pregnan* complication* or pregnan* adverse reaction* or pregnan* adverse effect* or fetal loss or fetal embryo loss or fetal death or embryo death or maternal mortalit* or maternal death or preconception care* OR prenatal care* OR postnatal care* OR postpartum care* OR maternal care* OR perinatal care* OR "post partum" care* OR "post natal" care* OR antenatal care* OR trimester* care* OR intrapartum care* OR preconception service* OR prenatal service* OR postnatal service* OR postpartum service* OR maternal service* OR perinatal service* OR "post partum" service* OR "post natal" service* OR antenatal service* OR trimester* service* OR intrapartum service* or preconception health OR prenatal health OR postnatal health OR postpartum health OR maternal health OR perinatal health OR "post partum" OR "post natal" health OR antenatal health OR trimester* health OR intrapartum health or ("still birth*" or stillbirth* or "chorea gavidarum" or "pregnancy associated choreoathetos*" or "pregnancy induced chorea*" or "gestational diabetes" or "pregnancy induced diabetes" or "pregnancy induced hypertension" or "pregnancy transient hypertension" or eclampsia* or pre-eclampsia* or "HELLP Syndrome" or "Hemolysis Elevated Liver Enzymes Lowered Platelets Syndrome" or "pregnancy toxemia*" or "Edema Proteinuria Hypertension Gestosis" or "nuchal cord" or "fetal cord entanglement*" or "ectopic pregnanc*" or "puerperal disorder*" or "placenta disease*"))

Year Range: 2000, 2020

| **Table S3** Conditions by Organ System | |
| --- | --- |
| **System** | **Disease conditions** |
| Allergy & Immunology | Allergy diseases  Dermatic allergies  Immune disorders  Major pinocytic disorders  Systemic allergies  Vasomotor and allergic rhinitis |
| Cardiovascular | Angina  Aortic dissection  Atrial fibrillation  Cardiac arrythmia  Cardiac conduction disorders  Cardiomyopathy  Chronic heart disease  Chronic hypertension  Dilated aortopathy  Intracerebral hemorrhage  Ischemic heart disease  Heart attack  Heart failure  Hypertensive disorders of pregnancy [gestational hypertension, pre-eclampsia]  Hypotension  Paroxysmal tachycardia  Peripartum cardiomyopathy  Pulmonary hypertension  Rheumatic heart disease  Valvular heart disease [incl. prosthetic heart valves and cardiac valve repair]  Severe left sided obstructive lesions  Severe systemic ventricular dysfunction  Stroke  Subarachnoid hemorrhage |
| Dermatology | Acne  Chronic ulcer of the skin  Dermatitis  Psoriasis  Rosacea  Urticaria |
| Ear, Nose and Throat | Chronic diseases of inner ear  Chronic diseases of middle ear and mastoid  Chronic diseases of the tonsils and adenoids  Chronic nasopharyngitis  Chronic pharyngitis |
|  | |
| **Table S3** Conditions by Organ System | |
| **System** | **Disease conditions** |
|  | Chronic rhinitis  Chronic sinusitis  Oral disease  Otitis externa |
| Endocrine | Gestational diabetes  Goitre  Impaired glucose tolerance  Hyperlipidemia  Hypothyroidism  Hyperthyroidism  Impaired fasting glucose  Obesity  Polycystic Ovary Syndrome  Pre-diabetes  Prolactinoma  Type I DM  Type II DM |
| Genetic/Hereditary | Congenital malformations  Deformations and conditions of the fetus originating in the perinatal period  Dwarfism  Genetic muscular disorder  Turner’s syndrome  Ehler’s Danlos  Marfan’s syndrome  Neurofibromatosis Type I  PKU  Spina bifida |
| Gastroenterology | Alimentary deficiencies  Chronic gastritis  Chronic diseases of liver, gallbladder and pancreas  Chronic liver disease  Cirrhosis  Crohn’s disease  Digestive disorder  Disorders of malabsorption  Gastric ulcer  GERD  Hepatic failure  IBS  Nausea and vomiting of pregnancy  Supernutrition  Ulcerative colitis |
|  |  |
| **Table S3** Conditions by Organ System | |
| **System** | **Disease conditions** |
| Gynecology | Abnormal or uterine vaginal bleeding  Chronic inflammatory disorders of the female genital tract  Chronic salpingitis and oophoritis  Fibroids  Infertility  Noninflammatory disorders of the female genital tract  Obstetric fistula  Ovarian dysfunction  Pelvic discomfort  Uterine prolapse |
| Hematology | Agranulocytosis  Antiphospholipid antibody syndrome  Coagulation defects  Factor V Leiden  Iron deficiency anemia  Protein C deficiency  Protein S deficiency  Pulmonary embolism and thromboembolic disease  Sickle cell disease  Thrombocytopenia  Thrombophilia |
| Infectious diseases | Hepatitis  Hepatitis B  HIV  HSV  Schistosomiasis  Sexually transmitted infections  Tuberculosis |
| Malignancy | Acute myeloid leukemia  Brain tumour  Breast cancer  Cervical cancer  Endometrial cancer  Glial tumour  Leukemia  Liver cancer  Lymphoma  Malignant melanoma  Ovarian cancer  Skin cancer  Trophoblastic disease  Uterine sarcoma |
|  |  |
| **Table S3** Conditions by Organ System | |
| **System** | **Disease conditions** |
| Mental Health | Adjustment disorder  Aggression  Anxiety  Autism  Bipolar disorder  Borderline personality  Delusional disorder  Depression  Depressive adjustment disorder  Dysthymic disorder  General mental distress  Panic disorder  Personality changes  Postpartum depression  Postpartum psychosis  Posttraumatic stress disorder  Psychotic disorder  Schizophrenia  Self-harm  Separation anxiety  Substance use disorder  Suicidal ideation |
| Musculoskeletal | ACL laxity  Back aches [incl. low back pain]  Carpal tunnel syndrome  Chondropathies  Collagenosis  Degenerative spondylolisthesis  DeQuervain’s tenosynovitis  Dorsopathies  Fractures  Hand pain  Internal derangement of the knees  Intervertebral disc disorders  Pubic symphysis diastasis  Rectus diastasis |
| Nephrology | Albuminuria  Chronic renal disease  Hyponatremia  Glomerular diseases  Kidney transplant  Nephropathies  Renal disease  Renal failure |
|  |  |
| **Table S3** Conditions by Organ System | |
| **System** | **Disease conditions** |
|  | Renal insufficiency  Renal tubulo-interstitial diseases |
| Neurology | Cerebral palsy  Dementia  Disease of the cerebral nerves  Diseases of the PNS  Epilepsy  Migraines  Mononeuropathy  Multiple sclerosis  Myopathies and paresis  Nerve pain  Seizure disorder  Spinal cord injuries  Upper extremity neuropathies |
| Ophthalmology | Chronic disease of the iris and ciliary body  Chronic disease of the retina  Disorders of accomodation and refraction  Disorders of optic nerve and visual pathways  Glaucoma  Other visual disturbances |
| Pulmonology | Asthma  COPD  Chronic respiratory disease  Pulmonary alveolar proteinosis  Pulmonary hemosiderin |
| Rheumatology | Ankylosing spondylitis  Fibromyalgia  Gout  Lupus  Osteoarthritis  Mixed connective tissue disease  Psoriatic arthritis  Rheumatoid arthritis  Scleroderma |
| Urology | Bladder disorders  Chronic cystitis  Urinary incontinence  Urolithiasis |
| Other | Bariatric surgery  Cesarean section  Childhood maltreatment  Chronic fatigue  Chronic pangs  Chronic physical disability  Chronic undernourishment  Complications of anesthesia  Emotional abuse  Malnutrition  Micronutrient deficiencies  Paternal factors  Permanent injuries from accidents  Physical abuse  Rape  Sexual abuse  Transplant |
